# Targeting Cellulite as a Metabolic–Dermal Interface: Effects of Tirzepatide and a Multi-Pathway Topical Therapy (G21) in a 12-Month Observational Study

**DOI:** 10.7759/cureus.107073

**Published:** 2026-04-15

**Authors:** Nikos Adamidis, Sofia Adamidi, Vasiliki E Georgakopoulou, Sotirios Adamidis

**Affiliations:** 1 First Department of Internal Medicine, Sismanoglio General Hospital, Athens, GRC; 2 Department of Internal Medicine, Charlton Memorial Hospital, Massachusetts, USA; 3 Department of Pathophysiology/Pulmonology, Laiko General Hospital, Athens, GRC; 4 First Department of Internal Medicine, Athens Medical Group, Athens, GRC

**Keywords:** adipose tissue, cellulite, metabolic–dermal interface, tirzepatide, topical therapy

## Abstract

Background

Cellulite is a highly prevalent condition associated with alterations in adipose tissue architecture, microcirculation, and connective tissue integrity. Emerging evidence suggests that cellulite may represent a manifestation of both local structural changes and systemic metabolic dysfunction. This study aimed to evaluate the independent and combined effects of systemic metabolic therapy with tirzepatide and a multi-target topical formulation (G21) on cellulite severity and body composition.

Methods

This retrospective observational cohort study included 92 consecutively treated participants with grade II-III cellulite. Participants were categorized into three groups according to treatment received in routine clinical practice: combination therapy (tirzepatide + G21), tirzepatide alone, or topical G21 alone. The primary outcome was the change in cellulite severity assessed using the Nürnberger-Müller classification scale over 12 months. Secondary outcomes included changes in body weight, waist circumference, and body composition parameters measured by bioelectrical impedance analysis. Between-group differences were assessed using one-way ANOVA, while correlations and multivariable linear regression were performed to evaluate predictors of treatment response.

Results

Significant improvements in cellulite severity were observed across treatment groups (mean change: −1.63 ± 0.81; p < 0.001), with the greatest reductions observed in the combination and topical therapy groups compared with tirzepatide monotherapy (p = 0.001). Weight loss differed significantly between groups (p = 0.007), with the largest reductions observed in the combination and tirzepatide groups. Importantly, changes in cellulite severity were not significantly correlated with changes in body weight (r = 0.059, p = 0.587) or fat mass (r = −0.115, p = 0.575). In multivariable analysis, baseline body weight (β = 0.811, p < 0.001) and baseline cellulite severity (β = −0.440, p = 0.015) were independent predictors of improvement, whereas weight loss and fat mass reduction were not.

Conclusions

Improvement in cellulite severity was not significantly associated with weight loss in this cohort and is more strongly associated with baseline tissue characteristics and targeted local interventions. While tirzepatide effectively reduces body weight, topical therapy appeared to be associated with a greater improvement in modulating cellulite-specific structural changes. The combination approach provides the most comprehensive benefit, supporting a potential complementary effect between systemic metabolic and local tissue-targeted mechanisms. These findings reinforce the concept of cellulite as a metabolic-dermal interface and support the use of integrated therapeutic strategies.

## Introduction

Cellulite is a highly prevalent cosmetic condition characterized by a dimpled, uneven skin surface, most commonly affecting the thighs, buttocks, and abdomen, with an estimated prevalence of up to 80-90% among post-pubertal women [1,2]. Although traditionally considered a benign aesthetic concern, increasing evidence suggests that cellulite reflects complex alterations in subcutaneous adipose tissue architecture, connective tissue remodeling, and microcirculatory dysfunction [3,4]. Histologically, cellulite is associated with hypertrophy of adipocytes, fibrotic thickening of fibrous septae, and impaired dermal-subcutaneous interactions, leading to the characteristic “orange-peel” appearance [3,5].

In parallel, metabolic dysfunction, particularly within the spectrum of metabolic syndrome (MetS), has emerged as a potential contributor to cellulite pathophysiology. MetS, defined by insulin resistance, central obesity, dyslipidemia, and hypertension, is associated with chronic low-grade inflammation, endothelial dysfunction, and adipose tissue dysregulation [6,7]. These mechanisms overlap significantly with those implicated in cellulite development, including adipocyte hypertrophy, extracellular matrix degradation, and impaired microcirculation [4,8]. Recent evidence supports a shared biological background between these conditions, suggesting that cellulite may represent not only a localized cosmetic issue but also a peripheral manifestation of systemic metabolic alterations [3,7-9].

Among metabolic pathways, insulin resistance plays a central role in both systemic and localized adipose tissue dysfunction. Hyperinsulinemia promotes lipogenesis, inhibits lipolysis, and contributes to adipocyte enlargement, thereby exacerbating subcutaneous fat protrusion into the dermis [10,11]. Additionally, inflammatory mediators such as tumor necrosis factor-α and interleukin-6 contribute to connective tissue remodeling and fibrosis, further worsening cellulite severity [7,12]. Microvascular impairment and lymphatic stasis, also observed in metabolic disorders, contribute to tissue edema and reduced clearance of metabolic byproducts, reinforcing the structural alterations characteristic of cellulite [4,13].

Tirzepatide, a novel dual glucose-dependent insulinotropic polypeptide (GIP) and glucagon-like peptide-1 (GLP-1) receptor agonist, has demonstrated significant efficacy in improving glycemic control, promoting weight loss, and reducing cardiometabolic risk factors [14,15]. Beyond its metabolic effects, tirzepatide may indirectly influence adipose tissue distribution, inflammation, and insulin sensitivity, factors that are mechanistically linked to cellulite pathophysiology. However, its potential role in modifying cellulite severity has not yet been systematically investigated.

In parallel, local and transdermal formulations targeting cellulite have focused on improving microcirculation, enhancing lipolysis, stimulating collagen synthesis, and reducing oxidative stress [16,17]. Despite advances in both systemic metabolic therapies and localized treatments, there remains a lack of longitudinal studies evaluating their independent and combined effects on cellulite using standardized and validated assessment tools. In particular, the potential complementary effect between systemic metabolic modulation (e.g., tirzepatide) and targeted topical formulations has not been explored.

Therefore, the present study aims to evaluate the effect of the combination of a new local formula and tirzepatide on cellulite severity over a one-year period, using standardized quantitative assessment methods. By integrating metabolic and dermatologic perspectives, this study seeks to provide novel insights into the treatment of cellulite as a condition influenced by both local and systemic factors. We hypothesized that (i) combined therapy would be associated with greater improvement in cellulite severity compared with monotherapy, and (ii) changes in cellulite severity would not be significantly associated with changes in body weight or fat mass.

## Materials and methods

Study design and population

This retrospective observational cohort study was conducted at the Lifestyle Medicine Department of Athens Medical Group and aimed to evaluate the effects of different therapeutic strategies on cellulite severity and body composition over a 12-month follow-up period.

The study population consisted of consecutively treated adult women aged 18-60 years who presented with clinically evident cellulite affecting the thighs and/or buttocks. Cellulite severity was assessed at baseline using the Nürnberger-Müller classification system [2] and was limited to grade II-III in all included participants to ensure a clinically homogeneous population. All patients had stable body weight, defined as a fluctuation of no more than ±3 kg during the three months preceding treatment initiation, and had not received any cellulite-targeted therapy within six months prior to baseline evaluation. No predefined BMI cut-off was applied for study entry; this was a real-world retrospective cohort of consecutively treated adults with grade II-III cellulite. The metabolic profile informed routine clinical decision-making, but diabetes, obesity, or metabolic syndrome were not mandatory inclusion criteria for the retrospective analysis.

Patients were excluded if they were pregnant, had active dermatologic conditions involving the treatment areas, presented with uncontrolled endocrine or metabolic disorders, or were receiving medications known to significantly influence adipose tissue metabolism or skin architecture, including systemic corticosteroids.

The study was conducted in accordance with the Declaration of Helsinki and was approved by the institutional ethics committee. Given the retrospective nature of the analysis, all data were derived from routine clinical practice and were anonymized prior to analysis.

Treatment exposure

Participants were classified into three groups according to the therapeutic approach received as part of routine clinical management. Treatment allocation was not randomized but was determined by individualized clinical decision-making based on patient characteristics, metabolic profile, and treatment preferences.

One group received combination therapy consisting of systemic metabolic treatment with tirzepatide alongside topical application of the G21 formulation. A second group received tirzepatide monotherapy, while a third group received topical G21 therapy alone. This real-world allocation allowed for comparative evaluation of systemic, local, and combined therapeutic approaches. Tirzepatide was prescribed for approved metabolic indications within routine clinical practice, including obesity and/or metabolic dysfunction, where clinically appropriate, and not as an approved treatment for cellulite per se.

Interventions

Tirzepatide was administered once weekly for up to 12 months, initiated at 2.5 mg weekly and titrated according to tolerability and clinical response, with individualized maintenance dosing documented from routine records. 

The G21 topical formulation was applied to the affected areas, specifically the thighs and/or buttocks, following a standardized application protocol. Participants were instructed to apply the formulation twice daily, in the morning and evening, with each application lasting approximately three to five minutes per treated area to facilitate dermal penetration and enhance local microcirculation; no washing for 1-2 hours; duration 12 months. Patients were further advised to avoid washing the treated areas for at least one to two hours following application and to refrain from using additional topical products in the same regions during this interval.

Throughout the study period, participants were advised to maintain their habitual dietary patterns and physical activity levels in order to minimize potential confounding effects related to lifestyle modification. Treatment adherence was assessed through patient self-report during routine follow-up visits. 

Description of the G21 formulation

The G21 formulation (G21 the secret gel) is a multi-component topical preparation developed as part of the G21 cellulite removal medical protocol (CRMP), designed to target the underlying pathophysiological mechanisms of cellulite through a synergistic, multi-pathway approach. The formulation is positioned as a therapeutic topical agent with a primarily physicochemical mode of action, consistent with its intended classification as a Class IIa medical device under EU MDR 2017/745.

The G21 formulation is a multi-component topical preparation comprising 11 bioactive ingredients selected based on documented mechanistic and experimental evidence targeting key biological pathways implicated in cellulite pathophysiology, including chronic inflammation, adipocyte dysfunction, extracellular matrix remodeling, microcirculatory impairment, and oxidative stress. The formulation includes dihydromyricetin (0.1-1%), caffeine (1-5%), tocopheryl acetate (0.5-1%), and plant-derived extracts such as Palmaria palmata (0.5-2%), Centella asiatica (0.5-2%), Camellia sinensis (0.5-3%), Ilex paraguariensis (0.5-2%), Vitis vinifera (0.5-2%), Zingiber officinale (0.1-1%), Mangifera indica (0.1-1%), and Rosa canina (0.5-2%). These concentration ranges are consistent with those commonly reported in topical dermatologic formulations and reflect the functional role of each component within a multi-target system.

The formulation is designed to exert its therapeutic effect through a coordinated, multi-pathway mechanism. Specifically, components such as caffeine and methylxanthine-containing extracts enhance lipolysis via cyclic AMP-mediated pathways, while dihydromyricetin and polyphenol-rich extracts modulate metabolic signaling through AMP-activated protein kinase activation and suppression of inflammatory cascades. Concurrently, agents such as Centella asiatica and vitamin C-rich extracts support collagen synthesis and extracellular matrix integrity, whereas antioxidant compounds, including epigallocatechin gallate, proanthocyanidins, mangiferin, and tocopherol, provide protection against oxidative stress and microvascular dysfunction.

Importantly, the formulation follows a systems-based design in which multiple active components converge on shared biological pathways, producing complementary and potentially synergistic effects. As such, the overall therapeutic activity reflects the integrated action of the formulation rather than the isolated effect of individual ingredients. The G21 formulation is applied topically twice daily to the affected areas for the duration of the study period [17-21].

Dihydromyricetin functions as a central metabolic regulator through activation of AMP-activated protein kinase (AMPK), suppression of NF-κB signaling, and inhibition of matrix metalloproteinases, thereby modulating adipose tissue metabolism and extracellular matrix integrity [18]. Caffeine enhances lipolysis via phosphodiesterase inhibition and increased intracellular cyclic AMP levels, while also exerting vasoconstrictive and anti-edematous effects [17]. Complementary botanical extracts contribute additional mechanisms, including inhibition of adipogenesis, stimulation of collagen synthesis, endothelial stabilization, and improvement of microvascular perfusion [19].

The formulation incorporates a coordinated antioxidant network consisting of polyphenols (e.g., epigallocatechin gallate, proanthocyanidins, mangiferin), vitamin C derived from Rosa canina, and vitamin E (tocopheryl acetate), which collectively protect dermal and vascular structures from oxidative damage. Concurrently, connective tissue remodeling is supported through enhanced fibroblast activity and inhibition of collagen degradation [20,21].

Importantly, the G21 formulation follows a systems-based design, whereby multiple active components converge on shared biological pathways, producing synergistic and complementary effects that cannot be attributed to any single ingredient alone. This multi-target approach addresses cellulite as a manifestation of both local tissue alterations and systemic metabolic dysregulation.

Outcome measures

An exploratory aim was to examine whether the observed clinical effects are consistent with the concept of cellulite as a metabolic-dermal interface.

The primary outcome was the change in cellulite severity from baseline to 12 months, assessed using the Nürnberger-Müller classification scale. The Nürnberger-Müller scale classifies cellulite into four grades (0-III) based on skin surface appearance at rest and after the pinch test [2]. Standardized digital images were obtained using consistent positioning, lighting conditions, and camera settings to ensure reproducibility.

Secondary outcomes included the changes of body composition parameters obtained by bioelectrical impedance analysis employing the BIA 101 device (Akern Srl, Florence, Italy), including total body water (TBW), extracellular water (ECW), fat-free mass (FFM), body cell mass (BCM), and fat mass (FM). Anthropometric parameters included body weight and waist circumference (WC).

For all variables, change values (D variables) were calculated as the difference between follow-up and baseline measurements.

Statistical analysis

Statistical analyses were performed using IBM Corp. Released 2020. IBM SPSS Statistics for Windows, Version 26. Armonk, NY: IBM Corp. Continuous variables were expressed as mean ± standard deviation (SD), while categorical variables were presented as frequencies and percentages.

Normality of data distribution was assessed using the Shapiro-Wilk test. For all outcome variables, change values (D variables) were calculated as the difference between follow-up and baseline measurements.

Baseline characteristics across the three treatment groups were compared using one-way analysis of variance (ANOVA) for continuous variables. Differences in outcome changes (D variables) between groups were also evaluated using one-way ANOVA. Where statistically significant differences were observed, post hoc analyses were performed. Post hoc comparisons indicated that the difference in cellulite improvement was primarily driven by the inferior response in the tirzepatide-only group compared with both the combination and topical therapy groups.

Associations between continuous variables were evaluated using Pearson correlation coefficients. Correlation analyses were specifically performed to assess the relationship between changes in cellulite severity and changes in body weight and fat mass.

To identify independent predictors of changes in cellulite severity, multivariable linear regression analysis was conducted. The dependent variable was the change in cellulite grade (D-cellulite), while independent variables included baseline characteristics (age, baseline body weight, and baseline cellulite severity) and changes in body composition parameters (D weight and D fat mass).

Homogeneity of variances was assessed using Levene’s test where appropriate. All statistical tests were two-tailed, and a p-value < 0.05 was considered statistically significant.

Ethics statement

The study was conducted in accordance with the Declaration of Helsinki and was approved by the institutional ethics committee of Athens Medical Group (Approval No.: 106/28-11-2024). Given the retrospective nature of the analysis, all data were derived from routine clinical practice and were anonymized prior to analysis.

## Results

Baseline characteristics

A total of 92 participants were included in the study. The mean age of the study population was 45.34 ± 11.01 years (range: 22-68 years). The cohort consisted predominantly of females (95.7%, n=88), while males accounted for 4.3% (n=4).

At baseline, significant differences were observed between the three treatment groups in terms of age, body weight, and waist circumference. No serious adverse events were identified in the medical records during follow-up.

Specifically, participants in the combination therapy group (Group 1) were significantly older compared to those receiving tirzepatide alone or topical treatment alone (47.72 ± 9.72 vs. 39.78 ± 10.63 and 39.95 ± 12.89 years, respectively; p = 0.006).

Body weight also differed significantly across groups (p < 0.001), with the highest mean weight observed in the tirzepatide-only group (89.44 ± 8.00 kg), followed by the combination group (81.58 ± 16.80 kg), while the topical-only group had a substantially lower baseline weight (66.90 ± 9.12 kg).

Similarly, waist circumference was significantly higher in the tirzepatide group (92.33 ± 16.14 cm) compared to the combination (87.30 ± 14.76 cm) and topical-only groups (76.00 ± 13.67 cm) (p = 0.006).

In contrast, no statistically significant differences were observed between groups in baseline cellulite severity (p = 0.652), indicating comparable initial disease burden across treatment arms.

Table 1 summarizes the baseline characteristics by treatment group.

**Table 1:** Baseline characteristics by treatment group. Baseline characteristics were compared across the three treatment groups using one-way analysis of variance (ANOVA). Test statistics are presented as F-values with corresponding degrees of freedom (df_between = 2, df_within = 89). Continuous variables are expressed as mean ± standard deviation (SD). A p-value < 0.05 was considered statistically significant.

Variable	Group 1 (Combination) n=64	Group 2 (Tirzepatide) n=9	Group 3 (Topical G21) n=19	Test Statistic (F) One way ANOVA	p-value
Age (years)	47.72 ± 9.72	39.78 ± 10.63	39.95 ± 12.89	F(2,89) = 5.37	0.006
Body Weight (kg)	81.58 ± 16.80	89.44 ± 8.00	66.90 ± 9.12	F(2,89) = 12.91	<0.001
Cellulite grade	2.82 ± 0.39	2.67 ± 0.52	2.79 ± 0.42	F(2,89) = 0.43	0.652
Total Body Water (L)	46.26 ± 8.76	45.34 ± 1.43	45.87 ± 7.36	F(2,89) = 0.03	0.967
Extracellular Water (L)	17.31 ± 3.97	18.80 ± 5.15	16.43 ± 5.71	F(2,89) = 0.44	0.648
Fat-Free Mass (kg)	58.23 ± 8.97	58.35 ± 5.03	59.18 ± 7.95	F(2,89) = 0.04	0.960
Body Cell Mass (kg)	37.64 ± 17.00	37.74 ± 20.16	35.72 ± 11.29	F(2,89) = 0.03	0.971
Waist Circumference (cm)	87.30 ± 14.76	92.33 ± 16.14	76.00 ± 13.67	F(2,89) = 5.36	0.006
Fat Mass (kg)	28.72 ± 12.80	32.48 ± 4.82	38.10 ± 4.24	F(2,89) = 0.41	0.662

**Figure 1:**
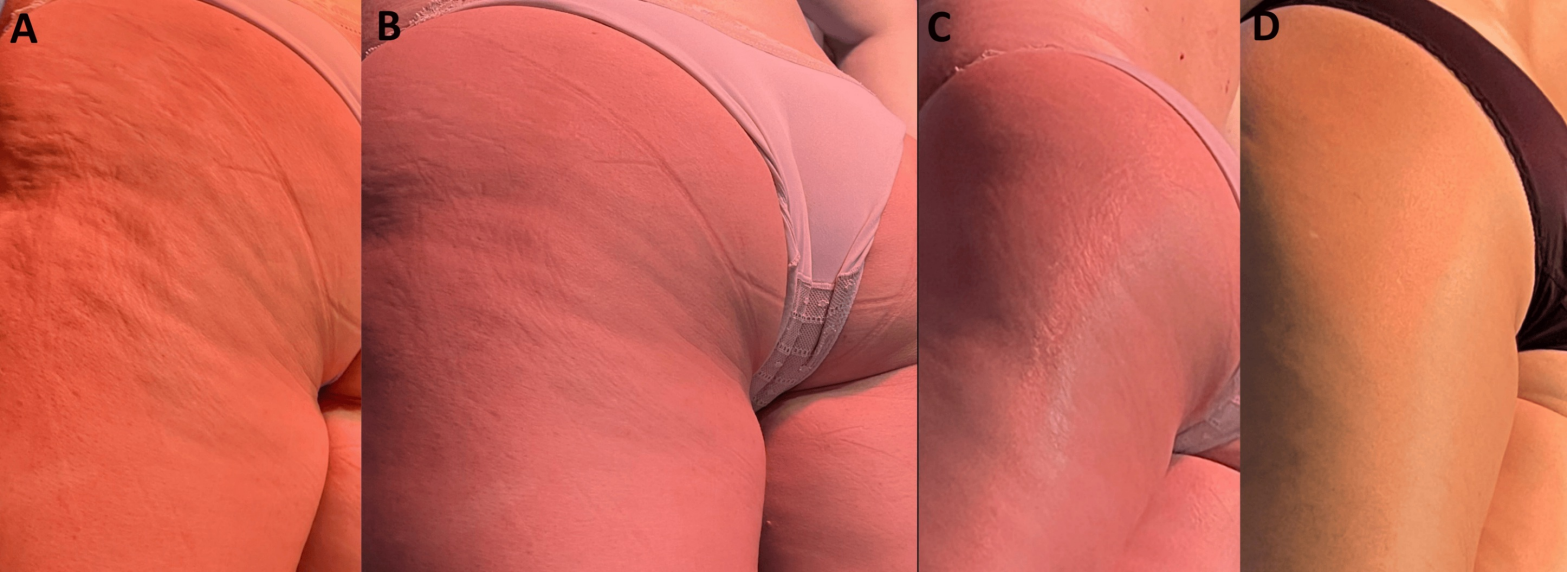
Images represent cellulite severity grades arranged from most severe to absent (grade III to grade 0). Representative standardized clinical images illustrating the four grades of cellulite severity arranged from severe to absent (left to right: Grade III to Grade 0). A. Grade III is characterized by pronounced skin dimpling with deep depressions visible in both standing and supine positions. B. Grade II presents a moderate “orange-peel” appearance visible without skin manipulation. C. Grade I demonstrates mild skin irregularities that are not visible at rest but may become apparent under certain conditions. D. Grade 0 corresponds to smooth skin without visible or palpable alterations.

Figure 1 illustrates the cellulite severity grades arranged from most severe to absent (Grade III to Grade 0).

Changes in clinical and body composition parameters

Significant differences between treatment groups were observed for changes in body weight and cellulite severity.

Specifically, weight reduction differed significantly across groups (p = 0.007), with the greatest decrease observed in the combination therapy group (−11.24 ± 8.67 kg), followed by the tirzepatide-only group (−10.89 ± 8.59 kg), while the topical-only group demonstrated a substantially smaller reduction (−4.34 ± 6.14 kg).

Similarly, improvement in cellulite severity differed significantly between groups (p = 0.001). The combination therapy group showed the greatest reduction (−1.73 ± 0.77), closely followed by the topical treatment group (−1.68 ± 0.75), whereas the tirzepatide-only group demonstrated a markedly smaller improvement (−0.50 ± 0.55).

No statistically significant differences were observed between groups for changes in total body water, extracellular water, fat-free mass, body cell mass, waist circumference, or fat mass (all p > 0.05).

Table 2 summarizes the changes in outcomes by treatment group.

**Table 2:** Changes in outcomes by treatment group. Changes in outcome variables (D values) were compared across the three treatment groups using one-way analysis of variance (ANOVA). Test statistics are presented as F-values with corresponding degrees of freedom (df_between = 2, df_within = 89). Continuous variables are expressed as mean ± standard deviation (SD). A p-value < 0.05 was considered statistically significant.

Variable	Group 1 (Combination)	Group 2 (Tirzepatide)	Group 3 (Topical G21)	Test Statistic (F) One way ANOVA	p-value
D Weight (kg)	-11.24 ± 8.67	-10.89 ± 8.59	-4.34 ± 6.14	F(2,89) = 5.18	0.007
D Cellulite grade	-1.73 ± 0.77	-0.50 ± 0.55	-1.68 ± 0.75	F(2,89) = 7.34	0.001
D Total Body Water (L)	3.59 ± 12.96	5.60 ± 5.26	2.79 ± 6.03	F(2,89) = 0.10	0.908
D Extracellular Water (L)	0.81 ± 5.33	0.20 ± 4.58	4.07 ± 6.82	F(2,89) = 1.12	0.329
D Fat-Free Mass (kg)	3.62 ± 11.84	7.22 ± 8.78	-0.81 ± 8.95	F(2,89) = 0.90	0.408
D Body Cell Mass (kg)	0.40 ± 17.76	0.27 ± 19.28	3.02 ± 8.12	F(2,89) = 0.05	0.949
D Waist Circumference (cm)	-10.98 ± 7.80	-8.33 ± 10.61	-7.76 ± 5.28	F(2,89) = 1.53	0.220
D Fat Mass (kg)	-14.15 ± 12.71	-16.58 ± 6.99	-28.90 ± 3.40	F(2,89) = 0.73	0.486

**Figure 2:**
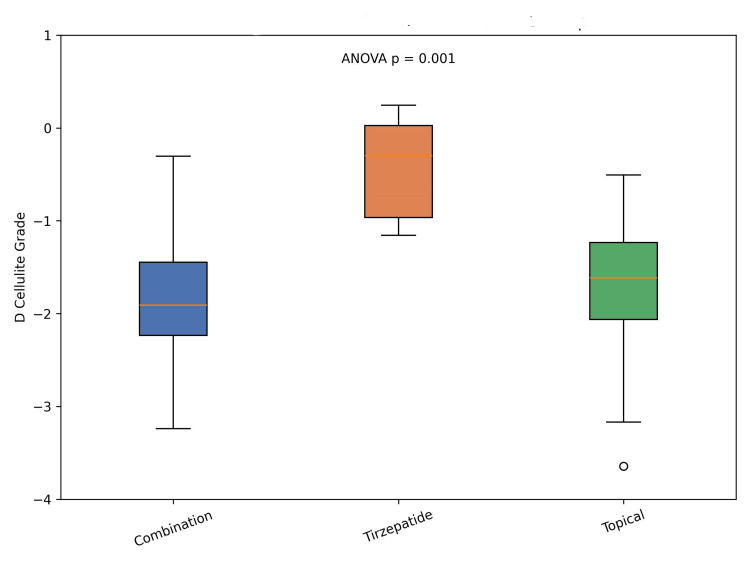
Change in cellulite severity across treatment groups. Box plots illustrate the distribution of changes in cellulite grade (D Cellulite) over 12 months for each treatment group (Combination, Tirzepatide, Topical). The central line represents the median, the box indicates the interquartile range (IQR), and whiskers extend to 1.5 × IQR. Individual points beyond this range are plotted as outliers. The isolated point observed in the topical group represents a statistical outlier, reflecting an individual with a markedly greater reduction in cellulite severity compared to the rest of the group. Overall group differences were assessed using one-way ANOVA (p = 0.001).

Figure 2 illustrates the change in cellulite severity across treatment groups.

Correlation analysis

Correlation analysis revealed no statistically significant associations between changes in cellulite severity and changes in body weight or fat mass.

Specifically, the change in cellulite grade was not correlated with weight reduction (r = 0.059, p = 0.587) or fat mass reduction (r = −0.115, p = 0.575).

Additionally, no significant association was observed between changes in body weight and fat mass (r = 0.301, p = 0.113).

These findings suggest that improvements in cellulite severity are independent of overall weight loss and fat mass reduction.

Multivariable regression analysis

A multivariable linear regression model was performed to identify independent predictors of changes in cellulite severity.

Baseline body weight emerged as a strong independent predictor of cellulite improvement (β = 0.811, p < 0.001), indicating that individuals with higher initial body weight experienced greater reductions in cellulite severity.

Additionally, baseline cellulite severity was independently associated with treatment response (β = −0.440, p = 0.015), suggesting that participants with more severe baseline cellulite demonstrated greater improvement.

In contrast, neither changes in body weight (p = 0.174) nor changes in fat mass (p = 0.505) were significantly associated with changes in cellulite severity.

Age was not a statistically significant predictor, although a trend toward association was observed (p = 0.099).

Table 3 displays the multivariable linear regression analysis for predictors of D-cellulite.

**Table 3:** Multivariable linear regression analysis for predictors of D-cellulite. Multivariable linear regression analysis was performed to identify independent predictors of changes in cellulite severity. Regression coefficients (B), standard errors (SE), standardized coefficients (Beta), and corresponding t-statistics are presented. The t-statistic was calculated as B/SE. A p-value < 0.05 was considered statistically significant. D refers to changes from baseline.

Variable	B	Std. Error	Beta	Test Statistic (t)	p-value
Constant	-3.246	1.068	-	-3.04	0.006
Weight (baseline)	0.042	0.009	0.811	4.67	<0.001
Age	0.020	0.012	0.274	1.67	0.099
Cellulite grade (baseline)	-0.913	0.345	-0.440	-2.65	0.015
D Weight	0.026	0.018	0.257	1.44	0.174
D Fat Mass	0.008	0.012	0.125	0.67	0.505

## Discussion

The present study demonstrates that both combined and individual therapeutic approaches targeting metabolic and local tissue pathways are associated with clinically meaningful improvements in cellulite severity over a 12-month period. Importantly, comparative analysis across treatment groups provides novel insight into the differential contributions of systemic metabolic modulation and localized therapy. While the combination of tirzepatide and topical treatment yielded the most favorable overall outcomes, distinct patterns of response were observed between groups, supporting a multifactorial model of cellulite pathophysiology.

A key finding of this study is that improvement in cellulite severity was not significantly associated with reductions in body weight or fat mass. Correlation analysis demonstrated no meaningful relationship between changes in cellulite grade and changes in body weight or adiposity, and these findings were further confirmed in multivariable regression analysis, where neither weight loss nor fat mass reduction independently predicted cellulite improvement. Instead, baseline characteristics, particularly initial body weight and baseline cellulite severity, emerged as the primary determinants of treatment response. These results challenge the widely held assumption that cellulite is primarily driven by adiposity and instead support the concept that cellulite represents a distinct structural and microenvironmental condition involving dermal-subcutaneous interactions, microvascular function, and extracellular matrix remodeling [1,22,23].

From a comparative perspective, tirzepatide monotherapy was associated with substantial weight loss but relatively modest improvement in cellulite severity, whereas topical therapy alone resulted in significant improvements in cellulite despite limited effects on body weight. The combination approach demonstrated both marked weight reduction and substantial improvement in cellulite, suggesting a potential complementary effect between systemic and local mechanisms. These findings reinforce the notion that while metabolic interventions may address upstream drivers such as adipocyte hypertrophy and systemic inflammation, targeted topical therapies are required to directly modulate local tissue architecture and dermal integrity [24,25].

The observed preservation and modest increase in fat-free mass further highlight the metabolic effects of the interventions, particularly in the context of incretin-based therapy. Maintenance of lean mass is critical for metabolic homeostasis and insulin sensitivity and may contribute indirectly to improved tissue function [15,26]. However, given the lack of association between changes in body composition parameters and cellulite improvement, these effects appear to play a secondary or supportive role rather than serving as primary drivers of the observed dermatologic outcomes.

The role of systemic metabolic modulation is particularly relevant in the context of tirzepatide, a dual glucose-dependent insulinotropic polypeptide and glucagon-like peptide-1 receptor agonist with well-established effects on weight reduction and insulin sensitivity [15,27]. Although these systemic effects may contribute to improvements in adipose tissue biology, endothelial function, and inflammatory status, the present findings suggest that such changes alone are insufficient to produce substantial improvements in cellulite. This dissociation underscores the importance of local structural factors, including connective tissue organization, microcirculation, and extracellular matrix dynamics, which are central to cellulite pathophysiology [1,22].

Conversely, the topical formulation appears to exert its effects primarily through local mechanisms, including enhancement of microcirculatory function, reduction of oxidative stress, and stimulation of collagen synthesis. Bioactive compounds such as caffeine promote lipolysis through phosphodiesterase inhibition, while plant-derived polyphenols exert antioxidant and endothelial-protective effects [17,28]. Additionally, agents such as Centella asiatica have been shown to stimulate fibroblast activity and collagen production, thereby improving dermal matrix organization and potentially reducing the appearance of skin depressions characteristic of cellulite [29]. The comparable magnitude of cellulite improvement observed in the topical and combination groups further supports the central role of local tissue modulation in the treatment of cellulite.

From a clinical perspective, these findings support a multimodal therapeutic strategy that integrates systemic metabolic optimization with targeted local interventions. Such an approach aligns with contemporary understanding of cellulite as a condition influenced by both systemic metabolic status and localized structural alterations. The demonstration that cellulite improvement is not solely dependent on weight loss has important implications for treatment strategies, particularly in individuals with normal or moderately elevated body weight, where purely metabolic approaches may be insufficient.

The present study has several strengths, including the use of a real-world clinical population, the comparative evaluation of three distinct therapeutic approaches, and the integration of clinical, anthropometric, and body composition data over a relatively long follow-up period. To our knowledge, this is among the first studies to systematically examine both the independent and combined effects of systemic metabolic therapy and a multi-target topical formulation on cellulite severity, highlighting a novel therapeutic paradigm that integrates local and systemic mechanisms.

Several limitations of the present study should be acknowledged. First, the retrospective and non-randomized design introduces the potential for selection bias and limits the ability to establish causal relationships between the interventions and the observed outcomes. Treatment allocation was based on individualized clinical decision-making in routine practice rather than predefined criteria, which may have resulted in baseline differences between groups that could influence treatment response despite statistical adjustment.

Second, although the composition of the G21 formulation, including the concentration ranges of its active components, is now reported and supported by established dermatologic and experimental literature, the formulation represents a multi-component, system-based intervention. As such, the independent contribution of each individual ingredient cannot be isolated within the context of this study, and the observed effects should be interpreted as reflecting the integrated activity of the formulation rather than the effect of specific components.

Third, while safety data were reviewed from available clinical records and no serious adverse events related to the interventions were identified, systematic and prospective adverse event collection was not performed due to the retrospective design. Therefore, the safety findings should be interpreted with caution, and rare or delayed adverse effects cannot be excluded.

Fourth, the study relied primarily on clinical grading scales and anthropometric measurements without the incorporation of advanced imaging modalities or molecular biomarkers. Consequently, direct assessment of structural and microenvironmental changes within the dermal and subcutaneous compartments was not feasible, and mechanistic conclusions remain inferential.

Fifth, although statistically significant associations were observed between treatment approaches and improvements in cellulite severity, the observational nature of the study precludes definitive conclusions regarding causality, mechanistic pathways, or interaction effects between systemic and topical therapies. Finally, residual confounding related to non-randomized treatment allocation cannot be excluded and may have influenced the observed differences between groups. Future prospective, randomized controlled studies incorporating standardized treatment protocols, objective imaging techniques, and biomarker-based assessments are required to validate these findings and further elucidate the underlying biological mechanisms.

Future studies should incorporate randomized controlled designs with larger and more balanced populations to validate these findings and further delineate the relative contributions of systemic and local therapies. The inclusion of advanced imaging techniques, such as high-resolution ultrasound or magnetic resonance imaging, would provide valuable insight into structural changes within the dermal and subcutaneous compartments. Additionally, integration of biomarkers related to inflammation, adipokine signaling, and microvascular function may help clarify the mechanistic pathways underlying treatment response.

## Conclusions

In this retrospective observational cohort, improvement in cellulite severity was observed across treatment groups and was greater in participants receiving topical G21, either alone or in combination with tirzepatide, than in those receiving tirzepatide alone. Changes in cellulite severity were not significantly correlated with changes in body weight or fat mass in this dataset. Because of the non-randomized design, baseline group differences, and limited objective outcome assessment, these findings should be interpreted as associative rather than causal and require confirmation in prospective controlled studies.
